# Long-term life history predicts current gut microbiome in a population-based cohort study

**DOI:** 10.1038/s43587-022-00286-w

**Published:** 2022-10-14

**Authors:** Jiyeon Si, Jorge F. Vázquez-Castellanos, Ann C. Gregory, Lindsey Decommer, Leen Rymenans, Sebastian Proost, Javier Centelles Lodeiro, Martin Weger, Marlene Notdurfter, Christoph Leitner, Peter Santer, Gregorio Rungger, Johann Willeit, Peter Willeit, Raimund Pechlaner, Felix Grabherr, Stefan Kiechl, Herbert Tilg, Jeroen Raes

**Affiliations:** 1 Department of Microbiology and Immunology, Rega Institute for Medical Research, Leuven, Belgium; 2 VIB-KU Leuven Center for Microbiology, Leuven, Belgium; 3 Medizinische Klinik II, Klinikum Ingolstadt, Ingolstadt, Germany; 4 Department of Internal Medicine, Hospital of Bruneck, Bruneck, Italy; 5 Department of Laboratory Medicine, Hospital of Bruneck, Bruneck, Italy; 6 Department of Neurology, Hospital of Bruneck, Bruneck, Italy; 7 Department of Neurology, Medical University Innsbruck, Innsbruck, Austria; 8 Clinical Epidemiology Team, Institute of Health Economics, Medical University of Innsbruck, Innsbruck, Austria; 9 Department of Public Health and Primary Care, University of Cambridge, Cambridge, UK; 10 Department of Internal Medicine I, Gastroenterology, Hepatology, Endocrinology and Metabolism, Medical University Innsbruck, Innsbruck, Austria; 11 VASCage, Research Centre on Vascular Ageing and Stroke, Innsbruck, Austria; 12 Present Address: Natural Product Informatics Research Center, Korea Institute of Science and Technology (KIST), Gangneung, Republic of Korea

**Keywords:** Microbiology, Machine learning, Ageing

## Abstract

Extensive scientific and clinical microbiome studies have explored contemporary variation and dynamics of the gut microbiome in human health and disease^1–3^, yet the role of long-term life history effects has been underinvestigated. Here, we analyzed the current, quantitative microbiome composition in the older adult Bruneck Study cohort (Italians, Bruneck, *n* = 304 (male, 154; female, 150); age 65–98 years) with extensive clinical, demographic, lifestyle and nutritional data collected over the past 26 years^4^. Multivariate analysis of historical variables indicated that medication history, historical physical activity, past dietary habits and specific past laboratory blood parameters explain a significant fraction of current quantitative microbiome variation in older adults, enlarging the explanatory power of contemporary covariates by 33.4%. Prediction of current enterotype by a combination of past and contemporary host variables revealed good levels of predictability (area under the curve (AUC), 0.78–0.83), with *Prevotella* and dysbiotic *Bacteroides* 2 being the best predicted enterotypes. These findings demonstrate long-term life history effects on the microbiota and provide insights into lifestyle variables and their role in maintaining a healthy gut microbiota in later life.

## Abstract (web summary)

The intestinal microbiome has an important role in health and disease; however, the long-term effects of lifestyle choices on microbiome alterations are incompletely understood. Here, based on extensive lifestyle and medical data collected over 26 years, Si et al. demonstrate that long-term life history can predict current enterotype in older adults.

## Main

The structure, function and dynamics of the human gut microbiome are generally studied in cross-sectional or short-term longitudinal settings. Contemporary microbiome variation is partially explained by host variables such as age, sex, stool consistency/transit time, health status, diet and medication^1^. However, the gut is a dynamic ecosystem, continuously perturbed by dietary intake and egestion or occasional exposures to medication and disease^5^. Isolated events and long-term lifestyle choices can permanently alter the microbiome^6^, yet long-term temporal effects have been understudied. While diet only allows future microbiome prediction up to 2 d after food consumption^7^, incomplete recovery of the original microbiota following antibiotic exposure even after 6 months implies that, when strong enough, perturbation effects can last long term^8^. As host health and lifestyle continuously impact the microbiome environment over time, a prospective collection of host data is necessary to study the long-term cumulative effects of life history, especially for long-lived human hosts.

Here, we capitalized on the community-based north Italian Bruneck Study cohort (*n* = 304 (male, 154; female, 150), age 65–98 years), which prospectively collected long-term, individualized host metadata (that is, food intake, lifestyle, medication, blood chemistry and clinical assessments) over 26 years (1990 to 2016) in 5-year intervals^4^. Fecal samples collected in 2016 from individuals aged 65–98 years were subjected to quantitative microbiota profiling (QMP), enabling association of current absolute microbiome abundances with historical metadata^9^. Using this unique dataset, we explored (1) the associations of historical variables and the current microbiome and (2) the predictive capacity of lifestyle history on the current microbiome.

**Fig. 1:**
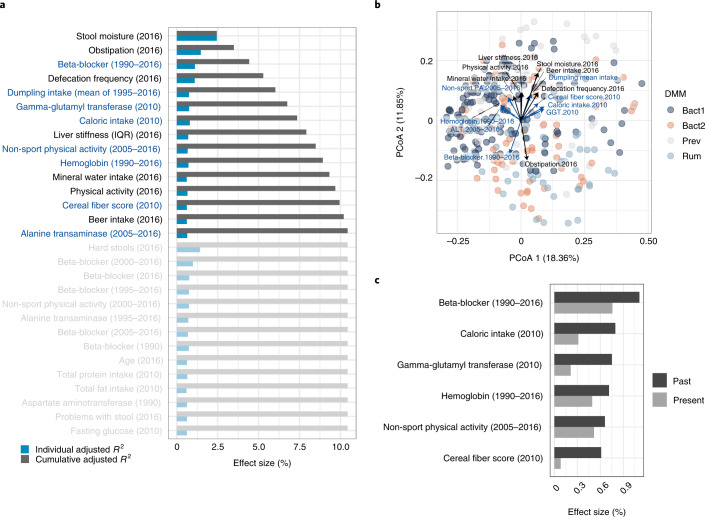
Explanatory variables for the microbiome variation in the Bruneck Study cohort. **a**, Individual and cumulative effect size of contemporary and historical covariates. Dark-colored bars indicate individual (upper bar) and cumulative (lower bar) effect sizes of variables included in the forward stepwise RDA model. Historical covariates are denoted with blue labels. IQR, interquartile range. **b**, Principal coordinate analysis (PCoA) based on Bray–Curtis dissimilarity. Arrows indicate significant covariates that can significantly explain the current microbiome variation. Colour key indicates different enterotypes. GGT, γ-glutamyl transferase; PA, physical activity. **c**, Comparison of the individual effect size of historical parameters and contemporary covariates. All statistical tests were performed on 304 individuals in the Bruneck Study cohort.

To first evaluate base explanatory power, we performed quantitative investigation of contemporary microbial community covariates using a distance-based redundancy analysis (db-RDA) approach as applied previously^1,2^. We identified 11 contemporary variables that could significantly explain the community variation with 7% nonredundant cumulative explanatory power. These analyses confirmed that covariates related to transit time (that is, current stool moisture, defecation frequency, hard stools and obstipation) contribute significantly to overall variations (db-RDA, adjusted *R*^2^ of 1.5–2.4%, false discovery rate (FDR) < 0.1, *n* = 304; Fig. 1a,b and Supplementary Table 1). We then assessed the potential of the extensive array of historical parameters collected during previous Bruneck Study evaluations (1990–2016) to explain current microbiome variation. Using historical parameters from each year as explanatory variables (Supplementary Table 2a), we identified several historical variables contributing significantly to a cumulative model that also included present variables (Supplementary Table 2b). Overall, significant historical variables were mostly linked to beta-blocker use, blood parameters and diet (db-RDA, adjusted *R*^2^ of 0.60–0.80%, FDR < 0.1, *n* = 304; Supplementary Table 2a). Interestingly, inclusion of these significant historical parameters significantly increased the cumulative nonredundant effect size to 8.5% (likelihood ratio test, *P* < 0.05; Supplementary Table 2b), indicating the potential explanatory power of long-term historical covariates on the current microbiome.

To better capture long-term lifestyle and health effects, we further investigated overall historical trends of variables using the average across all years and difference (that is, delta) for continuous variables and counts of event occurrence for categorical variables between each year and the year 2016. Analysis of averaged covariates revealed that only average intake of dumplings (canederli or knödel) from 1995 to 2016 was significant (db-RDA, adjusted *R*^2^ = 0.75%, FDR < 0.1, *n* = 304; Supplementary Table 3a). Given that canederli are traditional foods in the northeast region of Italy, this result is likely a proxy for a more traditional lifestyle. Covariate analysis of change (delta) in historical host parameters identified multiple non-colinear parameters independent of the time period covered (db-RDA, adjusted *R*^2^ = 0.63–1.11, FDR < 0.1, *n* = 304; Supplementary Table 3b and Fig. 1c). These were again analyzed with 11 significant contemporary covariates to calculate nonredundant cumulative effect sizes. Beta-blocker change from 1990 to 2016, non-sport physical activity change from 2005 to 2016, hemoglobin change from 1990 to 2016 and alanine transaminase change from 2005 to 2016 were shown to have significant explanatory power in addition to contemporary covariates, significantly raising the cumulative nonredundant effect size to 8.5% (likelihood-ratio test, *P* < 0.05; Supplementary Table 3c).

Finally, we combined all significant contemporary and historical features (Supplementary Tables 1a, 2a and 3a,b) in one comprehensive db-RDA analysis. This analysis found a final set of 15 variables significantly explaining the current microbiome variation. All together, they significantly increased the final cumulative nonredundant effect size to 10.4% (likelihood-ratio test, *P* < 0.05; Fig. 1a and Supplementary Table 3d). Overall, this shows that the inclusion of historical data resulted in a 33.4% increase in nonredundant explanatory power for global microbiota variation. To verify that the improvement in explanatory power was not due to just an additional number of data features but indeed reflects historical impact, we carried out an analysis in which we added all of the randomly permuted historical covariates to the 2016 data. The time effect was removed by using residuals obtained from autocorrelative models. These random features dropped the effect size to a lower level than with the results from only 2016 because the additional features served as nonsignificant covariates, increasing the multiple-testing correction hurdle and thus allowing fewer variables to enter in the selection model (cumulative nonredundant effect size of 4.36%; Supplementary Table 3e). These results confirm that the observed 33.4% increase in explanatory power is indeed attributable to historical covariates.

**Fig. 2:**
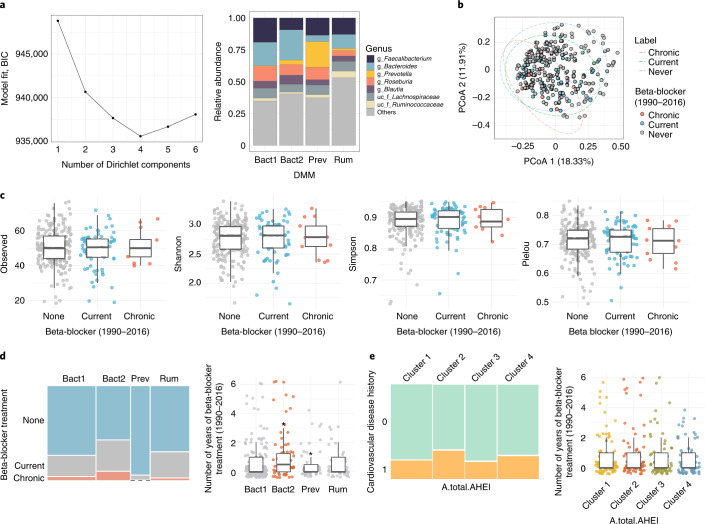
Association of beta-blocker history with microbiomes of older adults. **a**, Left, evaluation of model fit was performed using Bayesian information criterion (BIC) where the best model fit was found at four Dirichlet components. The FGFP cohort (*n* = 2,215) was used as a background dataset when enterotyping the Bruneck cohort. Right, top seven most abundant genera in enterotypes. **b**, Ordination plot by beta-blocker treatment (PCoA based on Bray–Curtis dissimilarity; Adonis *r*^2^ = 0.013, *P* = 0.0002). **c**, Biodiversity of individuals by beta-blocker treatment. No groups is significantly different. **d**, Left, prevalence of enterotype by beta-blocker treatment (Fisher’s exact test permuted, *P* = 0.0005). Chronic, treatment with beta-blocker both in 1990 and 2016; current, currently medicated; and none, not medicated in 1990 and 2016. Right, number of years of beta-blocker treatment across the years. An asterisk indicates FDR < 0.1 by Kruskal–Wallis test followed by post hoc Dunn’s test. **e**, Association of beta-blocker use with cardiovascular disease history and diet (chi-squared test, *P* = 0.327). Boxes represent the 25th percentile, median, and 75th percentile. Whiskers represent the lowest and highest values of the data. All statistical tests used were two sided and performed on 304 individuals in the Bruneck Study cohort. A.total.AHEL, total Alternate Healthy Eating Index.

We further deepened the relationship of these historical variables with the current microbiome by focusing on the current taxonomic group abundances as well as community enterotype based on Dirichlet multinomial mixtures (DMM) clustering previously validated across multiple cohorts^10–12^. Previous studies detected four enterotypes^9^, dominated by either *Bacteroides* (B1 and B2 enterotypes, with B2 having a lower microbial load and abundance of *Faecalibacterium* than B1)^13^, *Prevotella* (P) or Ruminococcaceae (R). All four enterotypes were present in the Bruneck cohort (Fig. 2a).

**Fig. 3:**
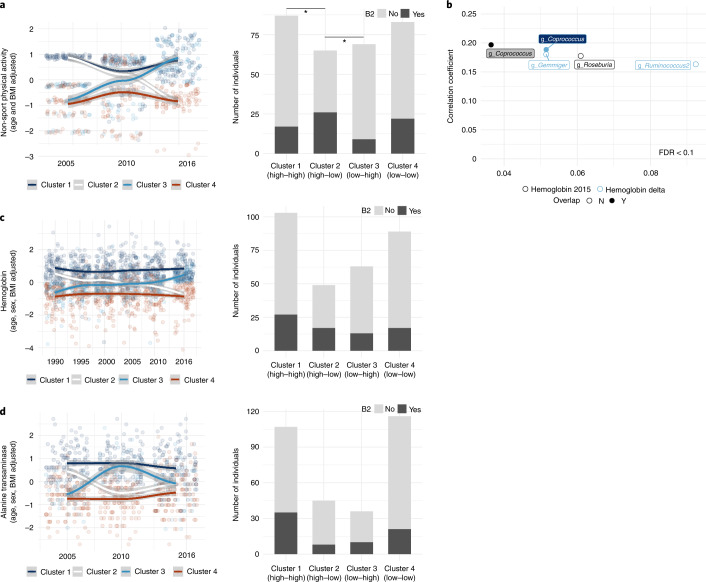
Link of life history with the gut microbiome of older adults. **a**, Left, clusters of non-sport physical activity across the years. Right, comparisons of the ratio of B2 and non-B2 by clusters were plotted by bar graphs. An asterisk indicates pairwise chi-squared test FDR < 0.1. **b**, Correlation of hemoglobin with current bacterial abundances after adjusting for age and stool moisture. Color-filled labels indicate taxa overlapping between historical and current levels of hemoglobin (partial correlation, FDR < 0.1). **c**,**d**, Comparison of clusters of hemoglobin (**c**) and ALT (**d**) across the years. Cluster 1, high activity in the past and at present; cluster 2, high activity in the past and low activity at present; cluster 3, low activity in the past and high activity at present; and cluster 4, low activity in the past and at present. All statistical tests used were two sided and performed on 304 individuals in the Bruneck Study cohort.

Of the significant historical covariates, we further analyzed beta-blocker treatment in association with community diversity. By dividing participants into three groups (chronic (treatment with beta-blocker both in 1990 and 2016), current (treatment with beta-blocker in 2016) and none (not medicated in 1990 or 2016)), we found that beta-blocker treatment was linked to a significant compositional shift (beta-diversity; Adonis *r*^2^ = 0.013, *P* < 0.001, *n* = 304; Fig. 2b and Supplementary Table 4a), but not to alpha-diversity (Kruskal–Wallis test, *P* > 0.05, *n* = 304; Fig. 2c). Enterotype prevalence was significantly different among the three groups (pairwise Fisher’s exact test, FDR < 0.1 for B2 and P versus other enterotypes, *n* = 304; Fig. 2d (left) and Supplementary Table 4b). In prticular, the B2 enterotype was more prevalent in individuals treated with beta-blocker than other enterotypes, whereas the P enterotype showed the opposite trend (Kruskal–Wallis test, FDR < 0.1, *n* = 304; Fig. 2d (right) and Supplementary Table 4c), with the former observation confirming previous findings^14^. Further analysis of specific taxonomic associations identified a list of bacteria more abundant in individuals who did not use beta-blockers, which can be potential targets for remediation strategies if future studies confirm a causal link for this association (generalized linear model (GLM), standardized *β* range of 4.3 to 0.78, FDR < 0.1, *n* = 304, adjusted for age and stool moisture; Supplementary Table 5). Additionally, we found a link between beta-blocker treatment duration and cardiovascular disease history (Wilcoxon test, *P* < 0.01; Supplementary Table 4d), but no associations with long-term dietary patterns, as determined by the total Alternative Healthy Eating Index (AHEI) (chi-squared test and Kruskal–Wallis test, *P* > 0.05; Fig. 2e). These results are in line with recent reports on the associations of microbiome changes with cardiovascular disease and beta-blocker use^14,15^. Analysis of average dumpling intake (1995–2016), a historical covariate with the second-largest effect size corresponding to an important staple food in this region, showed a significant association with *Dialister* abundance but not with enterotype (Spearman’s rho = 0.23, FDR < 0.1, adjusted for age and stool moisture, *n* = 304; Supplementary Table 6). We next looked at the change in non-sport physical activity between the years 2005 and 2016. We first identified taxa associated with both physical activity shifts (that is, the change from the past to the present) and current levels of physical activity. Although no genera were associated with both variables, butyrate-producing bacteria (that is, *Roseburia*, *Faecalibacterium* and *Butyricicoccus*) significantly increased in abundance with long-term physical activity (Spearman’s rho = 0.18–0.21, FDR < 0.1, adjusted for age and stool moisture, *n* = 304; Fig. 3b and Supplementary Tables 7 and 8). The positive influence of exercise on gut health has gained recent attention, with elevated abundance of *Roseburia* and *Faecalibacterium* reported in fit individuals and those who perform regular exercise^16–19^. To study the effects of changing physical activity, we clustered individuals into four categories: those with high activity in the past and at present (cluster 1), those with high activity in the past and low activity at present (cluster 2), those with low activity in the past and high activity at present (cluster 3) and those with low activity in the past and at present (cluster 4). Interestingly, individuals who had recently increased physical activity as well as those who had consistently maintained high activity exhibited a reduced ratio of (dysbiotic) B2 to non-B2 enterotypes. This suggests that physical activity has a beneficial role in the gut ecosystem of healthy older adults (pairwise chi-squared test, FDR < 0.1, *n* = 304; Fig. 3a and Supplementary Table 9).

Finally, we studied changes in hemoglobin between 1990 and 2016. Analysis of taxonomic association with both current hemoglobin and changes showed that another butyrate-producing bacterial genus, *Coprococcus*, was significantly associated with high levels of current hemoglobin as well as hemoglobin increase over time (Spearman’s rho = 0.19–0.20, FDR < 0.1, adjusted for age and stool moisture, *n* = 304; Fig. 3c and Supplementary Tables 10 and 11). This association could be linked to iron levels and/or consumption. For example, *Coprococcus* abundance was found to be lower in rats fed an iron-depleted diet and in infants with iron deficiency anemia^20,21^. At the enterotype level, the clustering approach used above did not show a significant association (Fig. 3c). Similarly, analysis of changes in alanine aminotransferase (ALT) between 2005 and 2016 showed that only the current ALT levels were significantly associated with *Methanobrevibacter* but not with enterotypes (Spearman’s rho = −0.18, FDR < 0.1, adjusted for age and stool moisture, *n* = 304; Supplementary Table 12 and Fig. 3d).

**Fig. 4:**
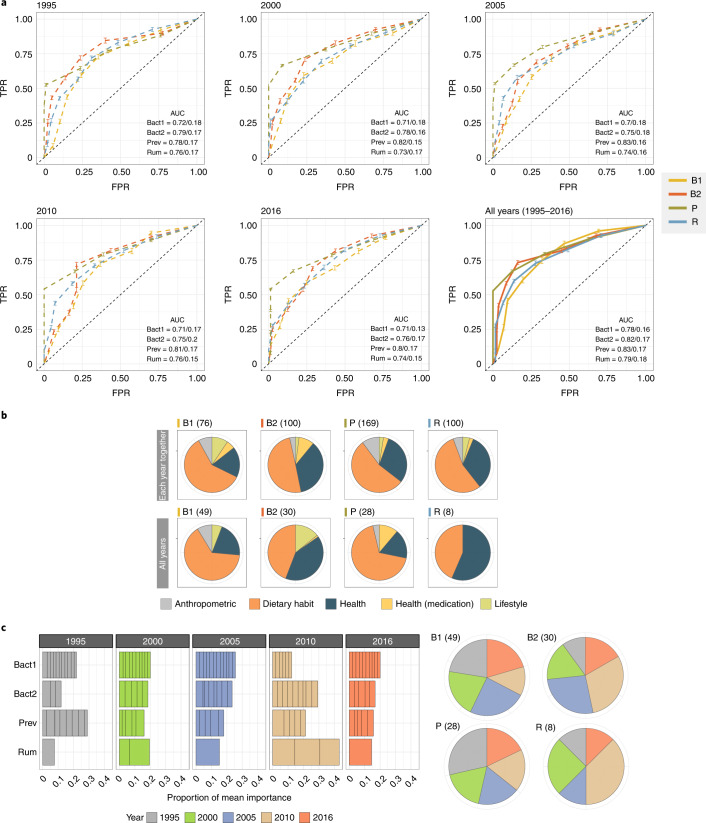
Prediction of current microbiome using life history. **a**, Receiver operating characteristic curve for the evaluations in 1995, 2000, 2005, 2010, 2016 and all years together based on 40 rounds of 40-fold cross-validation. Error bars indicate ranges of true-positive rate (TPR) in the cross-validation process. FPR, false positive rate. Data are shown as mean TPR ± standard error (SE) obtained from the cross-validation. The mean AUCs and their s.d. are shown in the bottom-right corner. **b**, Proportion of the variables in each category per enterotype. Top, variables selected from the analysis of each year. Bottom, variables selected from the analysis of all years together. **c**, Proportion of feature importance calculated for each enterotype in the analysis of all years together. Divisions within the bar chart indicate different variables. Values reported are the mean of the cross-validation replicates. The numbers in parentheses indicate the combined number of variables selected. All analyses were performed on 304 individuals in the Bruneck Study cohort.

**Extended Data Fig. 1:**
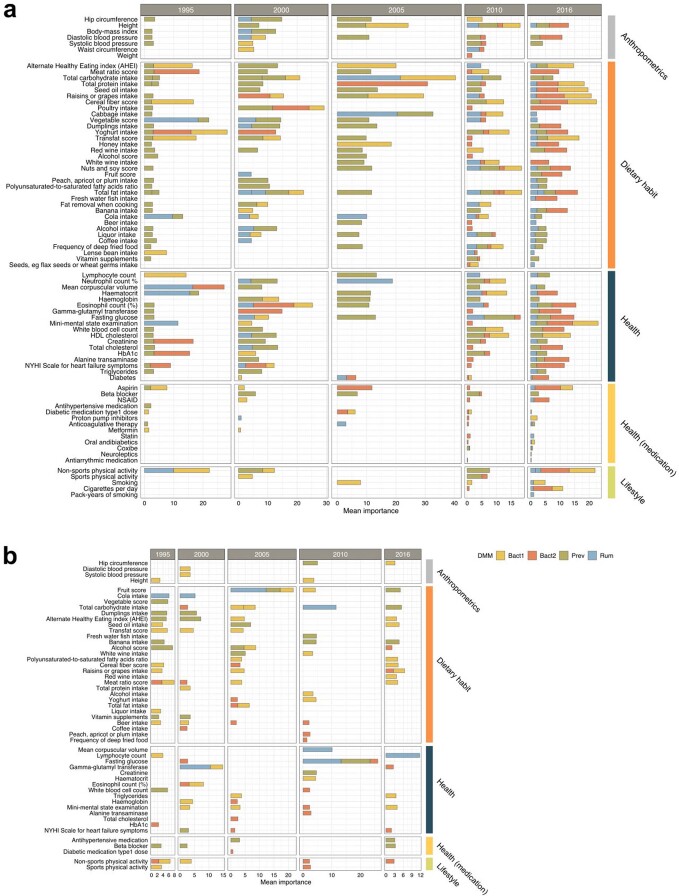
Prediction of current microbiome using life history. **(a)** Variables selected for enterotypes in the analysis of individual years and **(b)** all years combined. All statistical tests were performed on 304 subjects in the Bruneck Study cohort.

Next, we studied the predictive potential of life history on the current microbiome, moving from single-parameter models to more complex models. We first investigated long-term predictability by focusing on the power of the three significant individual historical variables from the year 2010 (db-RDA, FDR < 0.1, *n* = 304; Fig. 1a and Supplementary Table 3d) to predict current enterotypes, but no findings emerged (Kruskal–Wallis test, *P* > 0.05; Supplementary Table 13). Therefore, we sought to use a combination of variables as well as to investigate how far back we could use this combined information. To this end, we applied a random forest classifier with class balancing, feature selection and hyperparameter optimization (see Methods and Supplementary Information Fig. 2) to predict the current enterotype for each sampling year using only variables that were available across all years for parallel comparison. Models derived from a random training dataset were applied to test data using a *k*-fold cross-validation approach in an inner loop, which was repeated 40 times in an outer loop to estimate predictive power and avoid overfitting. Models performed well for all enterotypes each year with classification power highest for the P and B2 enterotypes (area under the curve (AUC) = 0.75–0.83; Fig. 4a). Interestingly, the prediction variables selected for each year showed distinct patterns for each enterotype (Fig. 4b, Extended Data Fig. 1a and Supplementary Table 14).

Finally, we built a prediction model using variables from all time points (Fig. 4a and Supplementary Table 15). As a result, we were able to improve the prediction power for all enterotypes based on the past and contemporary variables, yielding the best prediction level compared to all other years (AUC = 0.78–0.83). Prediction power was mostly found in variables from the diet and health categories (Fig. 4b and Extended Data Fig. 1b). The proportion of features selected was comparable between early (1995–2005) and recent (2010–2015) time points (Fig. 4c), but B1 and P presented more predictions from the early time points. Overall, these results suggest that past lifestyle variables can indeed predict the current microbiome.

**Extended Data Fig. 2:**
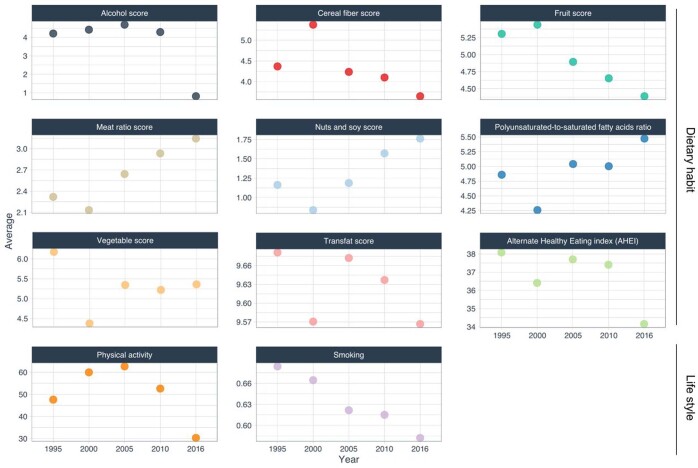
Changes in dietary habits and lifestyles across the years. Each dot indicates average value of the year. All statistical tests were performed on 304 subjects in the Bruneck Study cohort.

We performed multiple validations to verify these results. For instance, we tested whether suggested historical effects were confounded by consistency in lifestyle and diet throughout the years. Over the years, autocorrelation analysis of historical variables showed that only a few variables, such as vegetable score, liquor and seeds intake, shared a strong correlation (correlation coefficient > |0.5|) between the initial year and the first two time points (Extended Data Fig. 2 and Supplementary Table 16). Given that the autocorrelation could have lagged effects from previous time points, we further carried out a linear mixed model with the time points as the predictor and the historical variables as the dependent variables (Supplementary Table 17). These results also corroborated the observation that lifestyles and dietary patterns vary over the years (likelihood-ratio test, FDR < 0.1) and could thus contain relevant and different additional effects over contemporary data. Next, we permuted individuals and covariates of the residual matrix to study whether the improvement of prediction power observed with real historical data is replicated when an equal amount of random, but similarly structured, data is added. The results showed that, in all cases, predictions on real historical data outperform those with random data (Supplementary Table 18). These validations, combined with the extensive safety measures implemented in the model construction itself, minimize the chances that these models are overfitted. Thus, we provide evidence that the current gut microbiome is predictable by past variables and that inclusion of such variables can increase the variation explained by the current host lifestyle and physiology. Nonetheless, further validation in independent cohorts with a similar long-term sampling protocol would be warranted to confirm these results. Limitations of this study are the current lack of replication in similar large-scale long-term follow-up cohorts in other populations to assess the generalizability of these results. Additionally, use of shotgun metagenomics and meta-metabolomics could enhance taxonomic resolution and functional insights.

In conclusion, we show that an individual’s life history has long-term effects on the assembly of the gut microbiome. We report the predictability of the current gut microbiome by historical host parameters using a quantitative approach. Our results indicate that microbial community variation can be partly explained by the host’s life history. Specifically, we found that changes in an individual’s medication history, non-sport physical activity and hemoglobin levels over time were linked to the individual’s current microbiome. Further, we assessed the prediction potential of the historical metadata over the current microbiome composition and could predict an individual’s current enterotype based on the combination of past and contemporary host parameters. Overall, these results suggest that long-term history of host laboratory blood parameters, medication, diet and lifestyle can exert significant impacts on the current microbiome, highlighting the key variables that are important for maintaining a healthy gut at a later life stage.

## Methods

### Study cohort

The study protocol was approved by the ethics committees of Bolzano and Verona by Comitato Etico della Azienda Sanitaria dell’ Alto Adige, Provincia Autonoma di Bolzano, and conformed to the Declaration of Helsinki. Fecal samples were collected in the Bruneck Study, a prospective population-based study on the epidemiology and pathogenesis of atherosclerosis launched in 1990 in Bruneck in northwest Italy^6^. Bruneck is an urban area located in an alpine region in northern Italy (South Tyrol). The genetic background of the population is heterogeneous, with sizable segments of the population having either a northern Italian or Austro-German background. Population mobility within the Bruneck area is low, at 0.2% per year at the time of the study. The official population register contains information obtained from the national census and is continuously updated regarding births, deaths and changes of residence. The study population was recruited as a sex- and age-stratified random sample of all inhabitants aged 40–79 years (125 men and women each in the fifth to eighth decade of life) selected using a computer-based random number generator. The baseline examination was performed in 1990 (July to November) with follow-up evaluations at 5-year intervals. Of the total sample, 93.6% participated in the baseline examination. The study population was white. The study has extensive metadata on all individuals since 1990 with comprehensive evaluations every 5 years up to 2016. The 2016 evaluation was performed with a 6-month delay in the spring of 2016 rather than in the autumn of 2015 (as usual) due to delays in ethics approval. All study participants provided written informed consent. Stool samples (*n* = 325) were collected at the most recent time point during the 2016 evaluation when study participants were 65 to 98 years old. Metadata collected include anthropometric information, each individual’s physician-confirmed medical history and diseases, food intake, lifestyle, vascular risk factors, medication and laboratory parameters^4,22–26^. In the survey area, virtually all inhabitants are referred to one local hospital that works closely with the general practitioners, which allows retrieval of full medical information. Accordingly, in this study, information on clinical diseases (current and past) and morbidities as well as medication does not rely on the participant’s self-report but was validated by medical records and based on standard diagnostic criteria.

Dietary intake was evaluated by quinquennial (1995, 2000, 2005, 2010 and 2015) dietician-administered 118-item food-frequency questionnaires (FFQs) based on the gold-standard FFQ by Willett and Stampfer^27^ and adapted to the dietary peculiarities in the survey area^22,26^. Dieticians made use of illustrative photos of foods when exploring aphasic patients and of information provided by spouses, caregivers and nursing homes. For each item in the FFQ, a common unit or portion size was specified, and we instructed participants to customize how often on average they had consumed that amount in the past years. The nine response categories ranged from ‘never’ to ‘six or more times a day’. We calculated nutritional intake by assigning a weight proportional to the frequency of use for each food (once per day equals a weight of one), multiplying this weight by the nutrient value for the specified size and summing the contribution of all foods. Nutrient composition data for foods were based on the US Department of Agriculture Nutrient Database (Release 23) (US Department of Agriculture, Agricultural Research Service, 2010, USDA National Nutrient Database for Standard Reference, Release 23; http://www.ars.usda.gov/ba/bhnrc/ndl). We dissected complex foods into component foods using common recipes. Estimates of nutrient intakes were calorie adjusted. For this purpose, we used the residuals obtained by regressing polyamine or other nutrient intake on total energy intake^26,28^. The reproducibility and validity of the original FFQ are well documented^27^ and extend to its application in the Bruneck Study, in which it was compared against 9-d diet records^22,26^. The Alternative Healthy Eating Index (AHEI), a measure of diet quality, significantly associated with the risk of major chronic diseases in a large number of studies, was calculated as described previously^29^. We did not consider the ‘duration of multivitamin use component’ because multivitamin supplementation was almost absent in our cohort. Accordingly, this index has eight components in our study (vegetable score, fruit score, cereal fiber score, alcohol score, meat ratio score, nuts and soy score, trans-fat score, polyunsaturated-to-saturated fatty acids ratio)^29^. Physical activity was quantified using the Baecke questionnaire^30^ and the Adult Compendium of Physical Activities to rate activity intensities, and the average metabolic-equivalent hours per week were calculated using these results (overall and separated into sports and non-sport physical activity). Individuals were coded as current smokers or non-smokers (including former smokers) with assessment of pack-years of smoking^25^. Alcohol intake was quantified in grams per day. BMI was calculated as weight in kilograms divided by height squared in meters. Systolic and diastolic blood pressure measures were taken after the participant had been sitting for at least 10 min, and the mean of three independent measurements was calculated. Hypertension was defined as systolic blood pressure ≥140 mm Hg, or diastolic blood pressure ≥90 mm Hg or the use of antihypertensive drugs. Socioeconomic status was defined on a three-category scale (low, medium and high) based on information about the occupational status and educational level of the person with the highest income in the household. Blood samples were taken in the morning hours after an overnight fast and 12 h of abstinence from smoking and immediately processed or stored at −70 °C. Diabetes mellitus was diagnosed when fasting plasma glucose exceeded 126 mg dl^-1^ or when participants were on antidiabetic medication. Laboratory parameters were assessed by standard methods in certified laboratories as detailed previously^4,22–26^. All study participants underwent ultrasound and transient elastography (Fibroscan, Echosens) examination to evaluate hepatic steatosis and liver stiffness. Of 325 individuals, 20 were excluded because of missing data for laboratory parameters, liver stiffness, stool features and visceral fat thickness. Variables with missing data for fewer than five individuals were replaced by the cohort mean or data were otherwise removed throughout the analysis (variables removed: muscle mass (%), metabolic rate, Bristol stool score, and fat mass (kg)). The FGFP cohort used in the present study (*n* = 2,215) is an expanded version of the first round of sampling completed in 2014 (*n* = 1,106)^1,31^.

### DNA extraction and sequencing

Fecal DNA extraction and sequencing were performed as described previously^1^. Briefly, DNA was extracted from 150–200 mg of the frozen samples using the MagAttract PowerMicrobiome DNA/RNA KF kit (QIAGEN) following the manufacturer’s instructions. The V4 region of 16 S rRNA genes was amplified using the 515 F/806 R primer pair and purified using the QIAquick PCR Purification Kit. Sequencing was performed using the Illumina MiSeq platform (MiSeq Reagent Kit v2) and HiSeq 2500 system (151bp paired-end reads) for the Bruneck Study and the FGFP cohorts, respectively.

### Microbial load measurement by flow cytometry

Microbial load of the study cohort was measured as described previously^7^. Briefly, 200–250 mg of frozen (−80 °C) fecal aliquots was diluted in saline solution (0.85% NaCl; VWR International) and filtered using a sterile syringe filter (a pore size of 5 µm; Sartorius Stedim Biotech). Next, 1 ml of the microbial cell suspension obtained was stained with 1 µl of SYBR Green I (1:100 dilution in DMSO; Thermo Fisher Scientific) and incubated for 15 min in the dark at 37 °C. The flow cytometry analysis was performed using a C6 Accuri flow cytometer (BD Biosciences) according to Prest et al.^11^. Fluorescence events were monitored using the FL1 533/30-nm and FL3 > 670-nm optical detectors. The BD Accuri CFlow software was used to gate and separate the microbial fluorescence events on the FL1/FL3 density plot from the fecal sample background. A threshold value of 2,000 was applied on the FL1 channel. Based on the exact weight of the aliquots analyzed, cell counts were converted to microbial loads per gram of fecal material.

### Relative and quantitative microbiome profiling

After demultiplexing with LotuS v1.565 (ref. ^32^), fastq sequences were further processed following the DADA2 microbiome pipeline^33^. Briefly, sequence reads were first filtered and trimmed with the following parameters: truncQ=11, truncLen=c(130,200) and trimLeft=c(30, 30). Filtered reads were denoised using the DADA2 algorithm, which infers the sequencing errors. After removing chimeras, an amplicon sequence variant table was constructed, and taxonomy was assigned using the Ribosomal Database Project (RDP) classifier implemented in DADA2 (RDP trainset 16/release 11.5). The ELDERMET cohort data (*n* = 752) were obtained from the Sequence Read Archive under study accession number PRJNA283106. The dataset was processed using the same DADA2 pipeline following the recommendations for 454 sequencing technology and using the following filtering and trimming parameters: trimLeft=c(15) and truncLen=c(200). For the diversity analysis, we only included community-dwelling individuals and the first time point (*n* = 153).

To prepare the QMP table, the relative microbiome profiling (RMP) taxonomic table was then corrected for copy number and rarefied to even sampling depth by dividing the sequencing depth by the cell count and was subsequently multiplied by bacterial cell load to quantify the number of bacteria per gram of fecal sample as previously described in ref. ^9^. One participant was further excluded due to low read counts during the data conversion. Using this approach, the sequencing data became proportional to the microbial loads in the samples. All analysis was performed based on QMP unless otherwise noted.

### Fecal moisture content

Moisture content was determined as the percentage of mass loss after lyophilization from 200–300 mg of frozen aliquots of non-homogenized fecal material (−80 °C). Lyophilization was performed for 2 d.

### Fecal calprotectin measurement

Fecal calprotectin concentrations were determined using the fCAL ELISA kit (Bühlmann) on frozen fecal material (−80 °C). The level of calprotectin was corrected for the amount of fecal samples used.

### Microbiome and statistical analysis

Statistical and microbiome analyses were performed in R (version 3.6.0)^34^ using the phyloseq^35^, vegan^36^, pairwiseAdonis^37^, rcompanion^38^, CoDaSeq^39^, DirichletMultinomial^40^, lm.beta^41^ and ppcor^42^ packages. Past lifestyle and dietary patterns were tested by autocorrelation (function ‘acf’) and a linear mixed model followed by the likelihood-ratio test:

$$\begin{array}{l}{{{\mathrm{Null}}}}\,{{{\mathrm{model}}}}:{{{\mathrm{dietary}}}}\,{{{\mathrm{habit}}}}\,{{{\mathrm{or}}}}\,{{{\mathrm{lifestyle}}}}\sim \left( {1|{{{\mathrm{participant}}}}} \right)\\ {{{\mathrm{Alternative}}}}\,{{{\mathrm{model}}}}:{{{\mathrm{dietary}}}}\,{{{\mathrm{habit}}}}\,{{{\mathrm{or}}}}\,{{{\mathrm{lifestyle}}}}\sim {{{\mathrm{time}}}} + \left( {1|{{{\mathrm{participant}}}}} \right)\end{array}$$

For the microbiota associations with any host parameters, taxa found in less than 20% of the population were excluded for noise reduction and alleviation of multiple-testing correction. Comparison of two groups was performed using the Wilcoxon rank-sum test, and Kruskal–Wallis test was used when analyzing more than two groups followed by post hoc Dunn’s test. Count data were analyzed by Fisher’s exact test. Taxonomic associations with host parameters were determined by partial correlation to adjust for confounders using the R package ppcor^43^. All statistical tests used were two sided. All statistical tests were followed by multiple-testing correction using the Benjamini–Hochberg method when testing more than two features. Data distribution was assumed to be normal, but if this was not the case, nonparametric testing or data transformation was applied.

#### Analysis of community variations using the current and past variables

The explanatory power of cohort covariates and their combined effect size for the microbial community variation was evaluated as described previously^1^. Briefly, distance-based RDA (db-RDA) was performed on the genus level using the Bray–Curtis dissimilarity as implemented in vegan^36^. Covariates (FDR < 0.1) found in this step were entered for forward stepwise model selection to measure their cumulative effect sizes. Before the analysis, the collinearity of variables was assessed by using Spearman’s rank correlation and the Wilcoxon rank-sum test for continuous and binary variables, respectively. One of the collinear variables was removed based on its representativeness and the explanatory power of its effect size of > |0.8| (Supplementary Table 20). To assess the effect of past events or host parameter shifts on the current microbiome variation, different approaches were performed for continuous and binary variables (infection, medication and smoking). For continuous variables, variable shifts between each time point and the year 2016 were calculated by subtracting the values. History of the categorical binary variables was determined by summing the event that occurred between the two time points. Smoking was taken as smoking history if the individuals were current smokers at the time point. Comparison of past and present nonredundant effect size was performed by likelihood-ratio test.

#### Associations of the past with the current microbiome

Enterotyping based on the DMM approach was performed as described by Holmes et al.^43^ on a genus-abundance RMP matrix using the R package DirichletMultinomial^41^ and the FGFP cohort (*n* = 2,215) as a background dataset. Evaluation of model fit was performed using the Bayesian information criterion (BIC) where the best model fit was found at four Dirichlet components. Taxonomic association analysis after adjusting for age and stool moisture was performed by fitting a GLM (link = logit). Beta-blocker treatment and hemoglobin clusters were used as binary dependent variables and genera were used as independent variables. Standardized *β* coefficients were calculated using the R package lm.beta^41^. Significant associations of deconfounded genera with the host parameters were tested by performing likelihood-ratio tests. Clustering of individuals was carried out by categorizing them as high or low based on the median values measured in the first time point. Multiple linear regression was performed on non-sport physical activity, hemoglobin and alanine transaminase, regressing out the effect of age, sex and BMI. Before the regression, physical activity and alanine transaminase were transformed by inverse normal transformation to fit a normal distribution.

### Prediction of the current microbiome based on life history

To construct a microbiome prediction model, a random forest classifier (R package caret^44^) was trained by setting the historical metadata as the predictor variables and the enterotype as the response variable. Here, the historical covariates were corrected for time effects by retrieving residuals from autocorrelative models (that is, dependent variables ~ year) for each individual. Enterotype prediction was carried out for each time point and all years together to determine the most predictive variables regardless of the time points. We followed a nested cross-validation approximation, which includes data balancing, feature selection and hyperparameter optimization to eliminate redundant variables, simplify the model and improve the model’s performance. The outer loop was subjected to 40 rounds of *k*-fold cross-validation, while the inner loop was subjected to 5 rounds. Splitting the training dataset into training and validation datasets allowed for data balancing, feature selection and hyperparameter adjustment in the inner loop (Supplementary Information Fig. 2). The parameters that maximized the Matthews correlation coefficient (MCC), using function ‘mcc’ (R package mltools)^45^, and AUC values, using function ‘roc’ (R package pROC)^46^, were selected to train and test on the 40 partitions of the outer loop.

#### Data balancing

Due to the dataset’s imbalance property with the *Prevotella* enterotype showing the largest imbalance, a permuted covariate may be as good a predictor as the true historical data when one or more classes have meager proportions compared to the other classes. The enterotype distribution in the Bruneck cohort (B1 = 34.4%, B2 = 24.6%, P = 13.44% and R = 27.5%) was in the range of moderately (7:3) to highly (8:2 or 9:1) imbalanced. Enterotype data balancing was carried out using the synthetic minority over-sampling technique (SMOTE^47^) (R package DMwR^48^), the function ‘ROSE’ (R package ROSE^49^) and the down- and upsampling methods (R package caret^44^). To avoid overfitting due to the small sample proportion in the training partition, downsampling was skipped when the sample size was less than that in the first quartile (77 samples). As a result, four datasets were created, each of which was balanced independently. These datasets were further used for feature selection and hyperparameter tuning.

#### Feature selection

Feature selection was performed using recursive feature elimination (RFE)^44^. The RFE algorithm performs iterative modeling for feature selection. At each iteration, the top-ranked predictors are retained, and the model is reevaluated, with the best model being determined by the highest accuracy. This analysis was carried out using the function ‘rfe’ in the caret R package with the following parameters: functions=rfFuncs, method=‘cv’, metric=‘kappa’ and Number=10. The number of features selected from each iteration was set to be selected from one-quarter of the available covariates in the dataset. Cohen’s kappa metric was used as a selection criterion, given that it has a better performance than the accuracy score in imbalanced datasets. Feature selection was performed for each of the four previously balanced datasets (Supplementary Information Fig. 2).

#### Hyperparameter optimization

Once feature selection was performed, a random forest classifier was implemented for each of the four previously balanced datasets with its respective selected feature. Each model was tuned using a grid search optimization strategy (mtry=1:15 and ntree=1000 with 10 repetitions) using the caret R package^44^ functions ‘trainControl’ and ‘train’. The optimal parameters were the ones that had an AUC of > 0.7 and maximized the MCC, an index for an imbalanced dataset that incorporates all information from the confusion matrix.

#### Model performance

The model’s performance was assessed by applying the best parameters and features for each round to the remaining 40 rounds (functions ‘trainControl’*,* ‘train’ and ‘predict’; R package caret^44^). The model parameters and features that maximized the AUC and the mean MCC of the 40 rounds of *k*-fold cross-validation were selected as the best model. Random forest feature importance was estimated using the mean decrease in accuracy implemented in the caret package^44^ (function ‘varImp’).

#### Assessment of the effect of additional features

**Extended Data Fig. 3:**
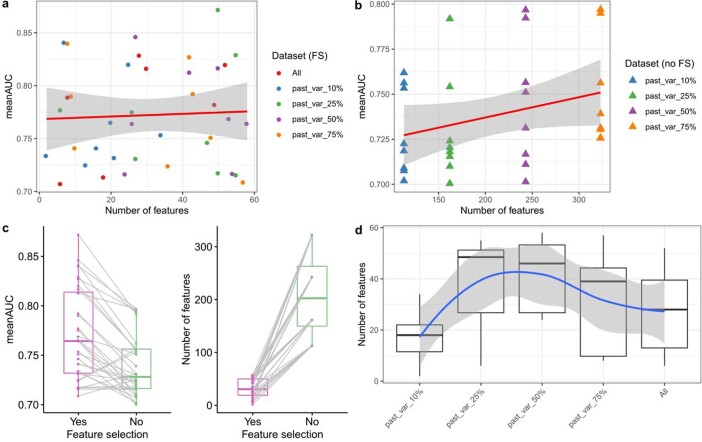
Evaluation of number of data features in modeling. **(a)** Relationship between number of features and AUC values in prediction models **(a)** with and **(b)** without feature selection. **(c)** Comparison of mean AUC and number of features between predictions with or without feature selection (Wilcoxon rank-sum test, p < 0.0001 for both). **(d)** Number of features entered in prediction models with feature selection (Spearman rho = 0.048; p−value = 0.771). Boxes represent the 25th percentile, median, and 75th percentile. Whiskers represent the lowest and highest values of the data. The grey bands represent the 95% confidence interval. All analyses were performed on 304 subjects in the Bruneck Study cohort.

To verify that the prediction based on covariates with data for all years was not solely due to increasing the size of the feature pool from which the model could select, we evaluated the effect of additional data features by adding an increasing numbers of randomly selected additional features (10%, 25%, 50%, 75% and 100% of the entire historical dataset) to the 2016 data. By comparing the mean AUCs of prediction models with and without feature selection, we observed that the additional number of features was not associated with greater prediction power (Spearman’s correlation, *P* > 0.05 (Extended Data Fig. 3a,b); of note, with feature selection, a smaller number of features enters the model compared to the initial input). AUC values were significantly improved with the feature selection approach, even with a lower number of features entering the model compared to the one without the feature selection (Wilcoxon rank-sum test, *P* < 0.0001; Extended Data Fig. 3c). Within the feature selection prediction models, a greater number of initial input features did not significantly increase the number of features entering into the prediction model (Spearman’s rho = 0.048; *P* = 0.771; Extended Data Fig. 3d).

### Statistics and reproducibility

We used all survival data from the Bruneck cohort since its inception in 1990; therefore, no statistical method was used to predetermine the sample size. Of 325 individuals, 20 were excluded due to missing data for laboratory parameters such as liver stiffness, stool features and visceral fat thickness. Missing data less than five was replaced by the cohort mean or otherwise removed throughout the analysis. To verify that the improvement in explanatory power was not due to an extra number of data features, we carried out the prediction analysis with randomly permuted historical covariates. The investigators were not blinded to allocation during experiments and outcome assessment.

### Reporting summary

Further information on research design is available in the Nature Research Reporting Summary linked to this article.

## Supplementary information

Supplementary Information Supplementary Figs. 1–3.

Reporting Summary

Supplementary Tables Supplementary Tables 1–20.

## Data Availability

Raw 16S data are available through managed access at the European Genome-Phenome Archive (https://ega-archive.org) under accession number EGAS00001004453. Data are available under controlled access for participant privacy reasons. They are available in accordance and in consent with ethical permission through managed access subject to a data use agreement with the FGFP and organized via principal investigator J.R. Derived species abundance counts and transformed microbial trait data can be found in Supplementary Table 19. Bruneck host metadata from this study are available in accordance and in consent with ethical permission through managed access and organized via principal investigator H.T. as follows: upon data request by email to herbert.tilg@i-med.ac.at, the Bruneck data, access committee will evaluate access permission, which will be granted upon signature of a data use agreement and material transfer agreement between the governing legal entities.

## Code availability

The custom script for the prediction analysis is available at https://github.com/raeslab/Enterotype-prediction-based-on-the-life-history.
