# Supplementary material for: Long-term life history predicts current gut microbiome in a population-based cohort study
Source: Nat Aging. 2022 Oct 14;2(10):885–95. doi: 10.1038/s43587-022-00286-w (PMC10154234; doi:10.1038/s43587-022-00286-w)
Supplement: Supplementary file 1 — Supplementary Figs. 1–3. [file 43587_2022_286_MOESM1_ESM.pdf]

nature aging

Letter https://doi.org/10.1038/s43587-022-00286-w

Long-term life history predicts current gut microbiome in a population-based cohort study

In the format provided by the authors and unedited

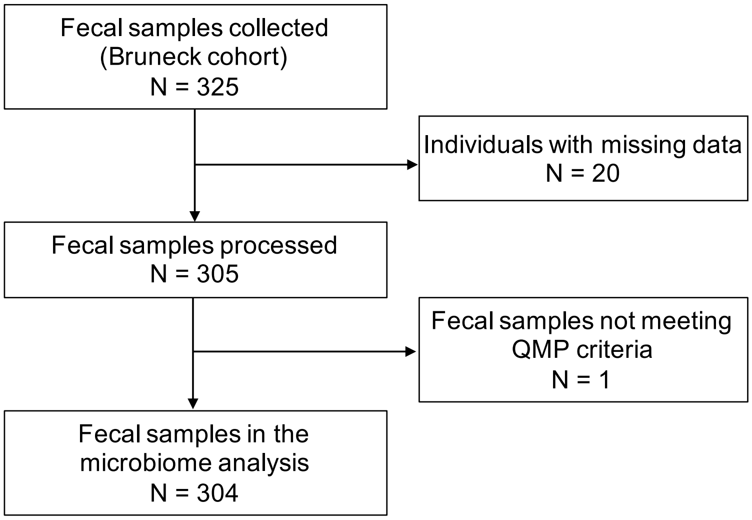

SI Figure 1. Schematic diagram of the Bruneck Study participant for the microbiome analysis.

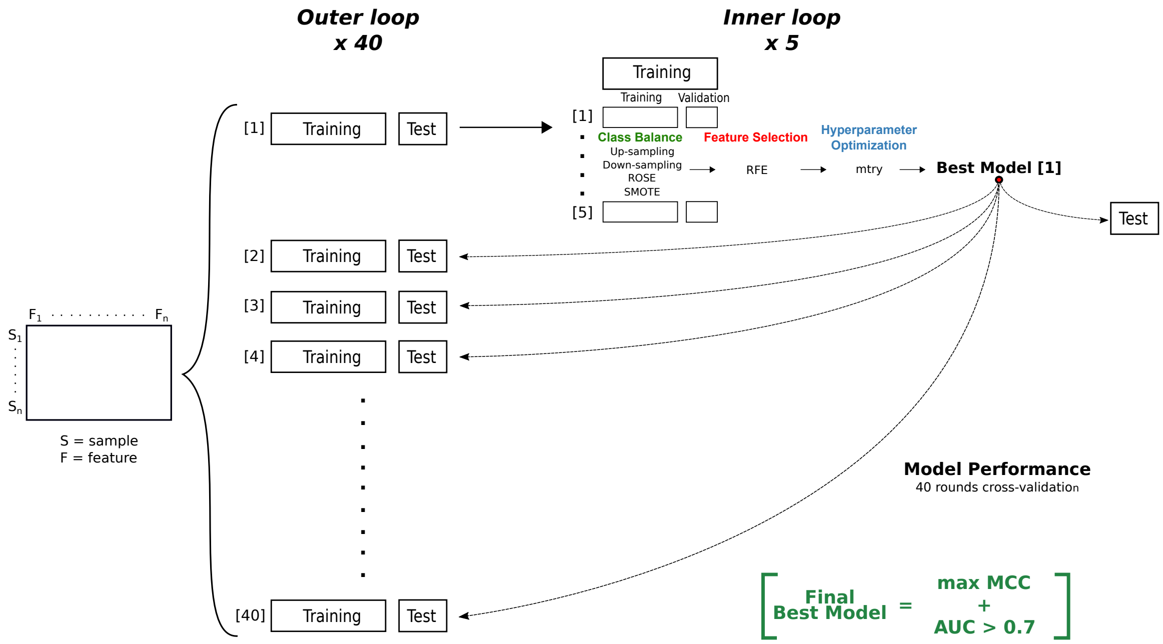

SI Figure 2. Schematic diagram of the nested cross-validation. The outer was subjected to 40 rounds of k-fold cross-validation while the inner loop was subjected to five rounds. Data balancing, feature selection, and hyperparameter tuning were carried out in the inner loop by splitting the training dataset into train and validation datasets. Parameters that maximize the Matthews correlation coefficient (MCC) and Area Under the Curve (AUC) values were used to train and test the remaining 39 partitions of the outer loop.

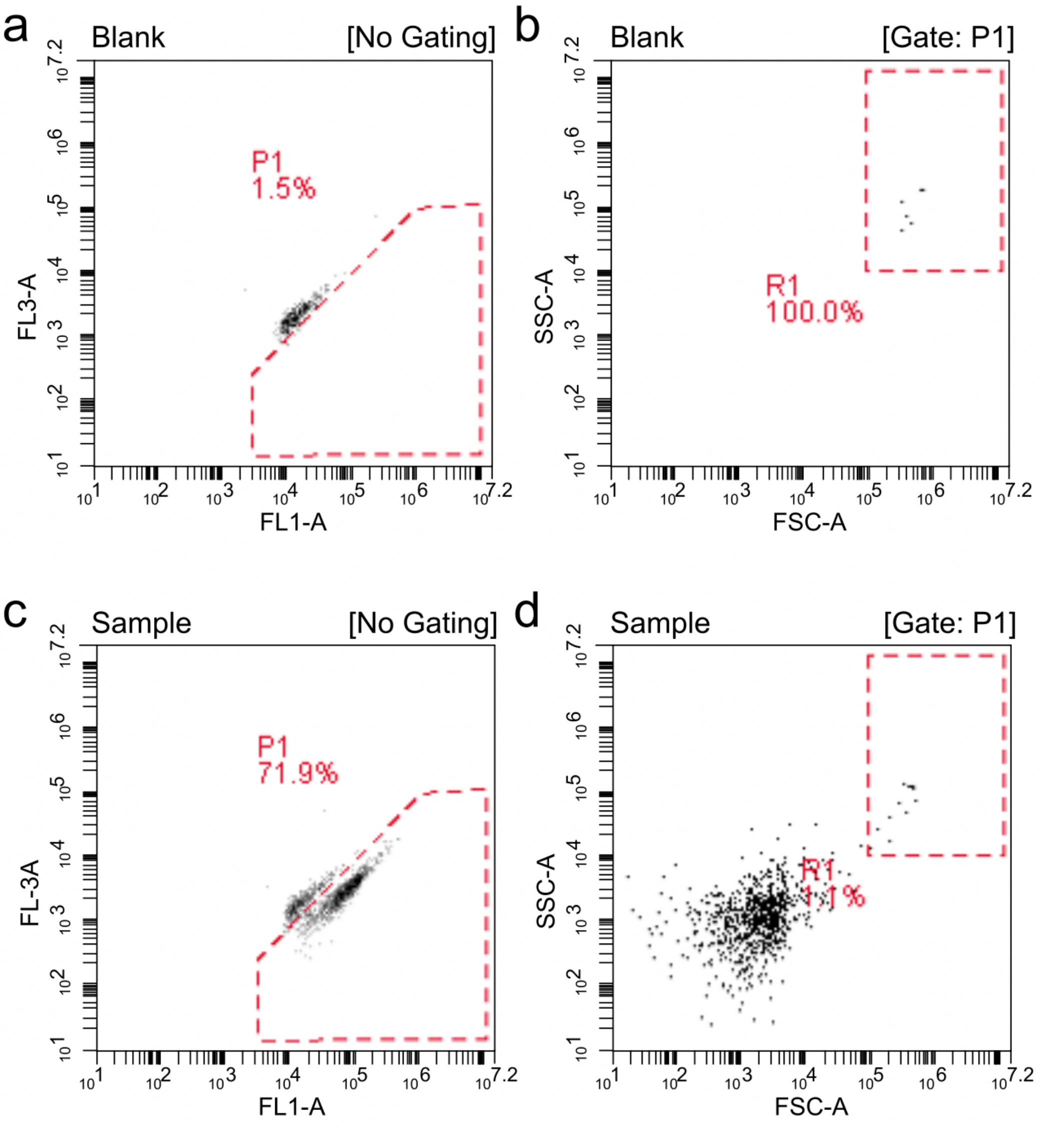

SI Figure 3. Flow cytometry gating strategy. A fixed gating/staining approach was applied[7]. Both blank and sample solutions were stained with SYBR Green I. (a) FL1-A/FL3-A acquisition plot of a blank sample (0.85% w/v physiological solution) with gate boundaries indicated. A threshold value of 2000 was applied on the FL1 channel. (b) Secondary gating was performed on the FSC-A/SSC-A channels to further discriminate between debris/background and microbial events. (c,d) FL1-A/FL3-A count acquisition of a faecal sample with secondary gating on FSC-A/SSC-A channels based on blank analyses. Total counts were defined as events registered in the FL1-A/FL3-A gating area excluding debris/background events observed in the FSC-A/SSC-A R1 gate. The flow rate was set at 14 microliters per minute and the acquisition rate did not exceed 10,000 events per second. Each panel reflects the events registered during a 30 seconds acquisition period. Cell counts were determined in duplicate starting from a single biological sample.
