# Supplementary material for: Long-term life history predicts current gut microbiome in a population-based cohort study
Source: Nat Aging. 2022 Oct 14;2(10):885–95. doi: 10.1038/s43587-022-00286-w (PMC10154234; doi:10.1038/s43587-022-00286-w)
Supplement: Supplementary file 2 — Reporting Summary [file 43587_2022_286_MOESM2_ESM.pdf]

nature portfolio

Corresponding author(s): Herbert Tilg, Jeroen Raes

Last updated by author(s): Jul 20, 2022

Reporting Summary

Nature Portfolio wishes to improve the reproducibility of the work that we publish. This form provides structure for consistency and transparency in reporting. For further information on Nature Portfolio policies, see our Editorial Policies and the Editorial Policy Checklist.

Statistics

For all statistical analyses, confirm that the following items are present in the figure legend, table legend, main text, or Methods section.

n/a	Confirmed	
☐	☒	The exact sample size (n) for each experimental group/condition, given as a discrete number and unit of measurement
☒	☐	A statement on whether measurements were taken from distinct samples or whether the same sample was measured repeatedly
☐	☒	The statistical test(s) used AND whether they are one- or two-sided<br>*Only common tests should be described solely by name; describe more complex techniques in the Methods section.*
☐	☒	A description of all covariates tested
☐	☒	A description of any assumptions or corrections, such as tests of normality and adjustment for multiple comparisons
☐	☒	A full description of the statistical parameters including central tendency (e.g. means) or other basic estimates (e.g. regression coefficient) AND variation (e.g. standard deviation) or associated estimates of uncertainty (e.g. confidence intervals)
☐	☒	For null hypothesis testing, the test statistic (e.g. F, t, r) with confidence intervals, effect sizes, degrees of freedom and P value noted<br>*Give P values as exact values whenever suitable.*
☒	☐	For Bayesian analysis, information on the choice of priors and Markov chain Monte Carlo settings
☐	☒	For hierarchical and complex designs, identification of the appropriate level for tests and full reporting of outcomes
☐	☒	Estimates of effect sizes (e.g. Cohen's d, Pearson's r), indicating how they were calculated

Our web collection on statistics for biologists contains articles on many of the points above.

Software and code

Policy information about availability of computer code

Data collection	Amplicon sequencing data were obtained using Illumina MiSeq platform. Flow cytometry analysis was performed using a C6 Accuri flow cytometer (BD Biosciences, New Jersey, USA) with BD Accuri Cflow software v.1.0.264.21 (BD Biosciences, New Jersey, USA).
Data analysis	Amplicon sequencing data were demultiplexed with LotuS (v1.565) and further processed following the DADA2 microbiome pipeline (R package version 1.12.1). Quantitative microbiome profiling (QMP) of microbiome data was prepared by correcting for copy number and rarefied to even sampling depth, which is the ratio of sequencing depth by the cell counts. Statistical and microbiome analysis were performed on Rstudio (v3.6.0) using phyloseq (v1.28.0), vegan (v2.5.6), pairwiseAdonis (v.0.0.1), rcompanion (v2.3.25), CoDaSeq (v0.99.4), DirichletMultinomial (v1.26.0), lm.beta (v1.5.1), and ppcor (v.1.1). Prediction analysis was carried out using Caret (v6.0-86), DMwR (v.0.4.1), ROSE (v.0.0-4), pROC (1.17.0.1), and mltools (0.3.5) packages.

For manuscripts utilizing custom algorithms or software that are central to the research but not yet described in published literature, software must be made available to editors and reviewers. We strongly encourage code deposition in a community repository (e.g. GitHub). See the Nature Portfolio guidelines for submitting code & software for further information.

Data

Policy information about availability of data

All manuscripts must include a data availability statement. This statement should provide the following information, where applicable:

- Accession codes, unique identifiers, or web links for publicly available datasets
- A description of any restrictions on data availability
- For clinical datasets or third party data, please ensure that the statement adheres to our policy

Raw 16S data is available through managed access at the European Genome/Phenome Archive (https://ega-archive.org) under accession number

EGAS00001004453. Data is available under controlled access for participant privacy reasons. It is available in accordance and in consent with ethical permission through managed access subject to a data use agreement with the FGFP and organized via principal investigator Jeroen Raes. Derived species abundance counts and transformed microbial trait data can be found in Supplementary Table 21. Bruneck host metadata from this study are available in accordance and in consent with ethical permission through managed access, and organized via Principal Investigator Herbert Tilg, as follows: Upon data request by email to Herbert.tilg@i-med.ac.at the Bruneck data access committee will evaluate access permission, which will be granted upon signature of a data use agreement/material transfer agreement between the governing legal entities.

Field-specific reporting

Please select the one below that is the best fit for your research. If you are not sure, read the appropriate sections before making your selection.

☒ Life sciences ☐ Behavioural & social sciences ☐ Ecological, evolutionary & environmental sciences

For a reference copy of the document with all sections, see nature.com/documents/nr-reporting-summary-flat.pdf

Life sciences study design

All studies must disclose on these points even when the disclosure is negative.

Sample size	No sample-size calculation was performed. A prospective cohort, the Bruneck study, started with 934 subjects in 1990 (Kiechl, 2019). After 26 years, we used the samples available for the Bruneck Study in 2016 (n = 325).
Data exclusions	Out of 325, 20 subjects were excluded due to missing data of laboratory parameters, liver stiffness, stool features, and visceral fat thickness. One subject was further excluded due to low read counts during the QMP data conversion (Supplementary Information).
Replication	Covariates with the highest effect sizes in explanation of the microbial community variation were successfully replicated in previous studies on the FGFP cohort (Falony et al. (2016) Science, Valles-Colomer et al. (2019) Nature Microbiology). For the prediction of the current microbiome, no prospective cohort with the past 26 years of metadata is available.
Randomization	The prediction analysis and db-RDA analysis were validated with a randomly permuted dataset. Given that this is not a case-control study, random allocation of experimental groups was not needed.
Blinding	Blinding is not relevant to this study as it is not an experimental study. This study is based on a prospective and population cohorts and a descriptive study.

Reporting for specific materials, systems and methods

We require information from authors about some types of materials, experimental systems and methods used in many studies. Here, indicate whether each material, system or method listed is relevant to your study. If you are not sure if a list item applies to your research, read the appropriate section before selecting a response.

Materials & experimental systems

n/a	Involved in the study	
☒	☐	Antibodies
☒	☐	Eukaryotic cell lines
☒	☐	Palaeontology and archaeology
☒	☐	Animals and other organisms
☐	☒	Human research participants
☒	☐	Clinical data
☒	☐	Dual use research of concern

Methods

n/a	Involved in the study	
☒	☐	ChIP-seq
☐	☒	Flow cytometry
☒	☐	MRI-based neuroimaging

Human research participants

Policy information about studies involving human research participants

Population characteristics	The Bruneck Study cohort includes 325 subjects recruited in Bruneck, northwest Italy (166 males and 159 females; age 65-98). Metadata (anthropometric information, physician-confirmed medical history and diseases, food intake, lifestyle, vascular risk factors, medication, and laboratory parameters) available for this study cohort can be found in the methods section.
Recruitment	The Bruneck Study, a prospective population-based study on the epidemiology and pathogenesis of atherosclerosis launched in 1990 in Bruneck. In the survey area, all inhabitants were referred to one local hospital that closely worked together with the general practitioners. Stool samples (n = 325) were collected at the most recent time point during the 2016 evaluation when study participants were 65 to 98 years old.
Ethics oversight	The study protocol was approved by the ethics committees of Bolzano and Verona by Comitato Etico della Azienda Sanitaria

Ethics oversight	dell´ Alto Adige, Provincia Autonoma di Bolzano and conforms to the Declaration of Helsinki. All study subjects provided written informed consent. No compensation was provided to participants for participatiing in the Bruneck Study.

Note that full information on the approval of the study protocol must also be provided in the manuscript.

Flow Cytometry

Plots

Confirm that:

☒ The axis labels state the marker and fluorochrome used (e.g. CD4-FITC).

☒ The axis scales are clearly visible. Include numbers along axes only for bottom left plot of group (a 'group' is an analysis of identical markers).

☒ All plots are contour plots with outliers or pseudocolor plots.

☒ A numerical value for number of cells or percentage (with statistics) is provided.

Methodology

Sample preparation	200-250 mg frozen (-80°C) faecal aliquots were dissolved in saline solution (0.85% NaCl; VWR International, Germany) to a total volume of 100 mL. This slurry was further diluted 1,000 times and filtered using a sterile syringe filter (pore size of 5 µm; Sartorius Stedim Biotech GmbH, Germany). Next, 1 mL of the microbial cell suspension obtained was stained with 1 µL SYBR Green I (1:100 dilution in DMSO; 10,000 concentrate, Thermo Fisher Scientific, Massachusetts, USA) and incubated for 15 min in the dark at 37°C.
Instrument	C6 Accuri flow cytometer (BD Biosciences, New Jersey, USA)
Software	BD Accuri Cflow software v.1.0.264.21 (BD Biosciences, New Jersey, USA).
Cell population abundance	Not applicable. No sorting of fractions was performed.
Gating strategy	Fluorescence events were monitored using the FL1 533/30 nm and FL3 >670 nm optical detectors. In addition, also forward and sideward-scattered light was collected. The BD Accuri CFlow software was used to gate and separate the microbial fluorescence events on the FL1/FL3 density plot from the faecal sample background. A threshold value of 2000 was applied on the FL1 channel. The gated fluorescence events were evaluated on the forward/sideward density plot, as to exclude remaining background events. Instrument and gating settings were kept identical for all samples

☒ Tick this box to confirm that a figure exemplifying the gating strategy is provided in the Supplementary Information.
